# An Account of the Extirpation of the Parotid Gland

**Published:** 1831

**Authors:** 


### WESTERN JOURNAL

OF THE

### MEDICAL AND PHYSICAL SCIENCES.

JANUARY, FEBRUARY, AND MARCH, 1831.

### ESSAYS AND CASES.

**Art. I.**—*An account of the Extirpation of the Parotid Gland.* In a letter from Professor McClellan to the Editors of the Western Journal.

*Philadelphia, Jan.* 30, 1831.

Gentlemen—

In the spring of the last year, I performed the operation of extirpating the parotid gland, an account of which was published in the 4th No. of the New-York Medical and Physical Journal, by Dr. Sorber, of this state, the brother of the patient. As that journal was discontinued, without the number in which Dr. Sorber's paper was published being generally distributed, and as the recent death and dissection of the patient have afforded materials for a complete and authentic history of the case, I have it proper to transmit you the whole for insertion in the Western Journal.

[The following is the entire article, as extracted from the New-York Journal.]

*A Case in which the right Parotid Gland, in a State of Melanotic Enlargement, was extirpated.* By George McClellan, M. D., Professor of Surgery in Jefferson College, Philadelphia, communicated by Joseph E. Sorber, M. D., of Pottsville, Pennsylvania.

In looking over a recent publication, (Tavernier's Surgery, translated by S. D. Gross, M. D.,) I found a note alluding to an operation which was performed on a brother of mine, and considering it, as I do, to be one of the most extraordinary attempts ever made to relieve a fellow mortal from inevitable death, I shall avail myself of this opportunity to place it more in detail before the medical public; not only as a tribute of respect to my friend, Dr. McClellan, but likewise as a case illustrating the practicability of removing an entire and enlarged parotid gland. It will at least decide a subject which has agitated the minds of many surgeons—some altogether denying its possibility, while others more intrepid pronounce it hazardous in the extreme, yet advisable under certain circumstances.

The present case was of about five years standing. I first saw the tumor in 1825, which then appeared to be situated immediately over the parotid gland. Not having time to operate, I advised the patient to call on his family physician and have it removed. It was neglected, however, from time to time, until the month of April, 1829, when, at the urgent request of my brother, I again visited him from my place of residence, (Pottsville, Schuylkill co., Pa.,) and was astonished to behold the increase of the tumor, and the ravages it had made on his constitution. I doubted the possibility of his being relieved from the hectic fever which had afflicted him for the preceding few months. He had, however been long troubled with a loss of appetite and difficulty of deglutition, and pain on the least motion of the jaw. I stated to him my ideas of his case, and my fears on the prospect of a cure, but at the same time advised a consultation with Dr. McClellan. In the consultation which followed, Dr. McClellan agreed with me in opinion, as to the dangers of the case, and declared that the sooner the enlargement could be removed the better. Accordingly the operation was soon after performed by him at my request.

He proceeded to the removal of the tumor, first by making a curvilinear incision on each side of it, commencing just above the zygoma, and extending down the neck until they met about two inches below the angle of the jaw. These were deepened until the massetre muscle and angle of the jaw were exposed in front, and the mastoid process and muscle behind the tumor. As the chief mass of the tumor appeared to extend below and behind the face, where it was firmly wedged down beyond the reach of all further dissection in that direction, the operator changed his mode of proceeding, and began to extend the incision about two inches further down the neck. This brought into view an oblong process of the tumor, about two inches in length, and three-fourths of an inch in diameter, which extended down from the main body of the gland, and was lodged in the common sheath of the great vessels. On raising this process of the tumor from below, the common carotid artery and jugular vein were fairly exposed, opposite the side of the thyroid cartilage. As the whole surface bled profusely, we concluded that it would be necessary to take up the carotid artery. It was accordingly secured at once, with a single ligature; immediately after which the hemorrhage subsided, and the exposed surface became perfectly dry.

Dr. McC. then proceeded to burrow under the body of the tumor, using the handle, and occasionally the blade of his scalpel. The back part of the tumor was then found to be attached to the transverse processes of the two upper cervical vertebræ, and to have become mbedded in, or incorporated with, the posterior belly of the digastric and the upper portions of the sterno-mastoid and trachelo mastoid muscles. These muscles were therefore cut away, in part, and the bones denuded; after which, the tumor was dissected upwards from underneath the ramus of the jaw and mastoid process: the styloid process being laid bare, and also the slender muscles which originate from it. In this part of the operation, the trunk of the *portio dura* was brought up from under the mastoid process, and of course divided along with the tumor. Several branches of the external carotid were also divided, two of which bled from the recurrent circulation, and had therefore to be secured; viz., the occipital and facial arteries.

The mass which had thus been elevated was evidently a morbid enlargement of the parotid gland, partially enveloped in a thin capsule, and composed of a black medullary substance, interspersed among the healthy looking granules of the parotid. The back, or under part of this mass had no capsule, and the deepest part of the exposed cavity, which corresponded with this, was occupied by a healthy looking portion of the gland, in wich a large branch of the nerve was seen ramifying. We agreed that it would be improper to leave this remnant of the gland behind. Dr. McClellan, therefore, dissected it out completely, and brought it up over the zygoma, along with the morbid part which he had before raised above the jaw. This last step was followed by an alarming gush of arterial blood, which most probably proceeded from the internal maxillary artery, but was speedily arrested by the tenaculum and ligature.

The whole mass, in one partly divided body, was finally dissected away from the zygoma, to which it was firmly tied by a tendinous fascia, during the division of which, the patient complained of more pain than in all the other stages of the operation. It appeared to me to be a remarkable circumstance that he evinced no pain when the *portio dura* was dissected up, although since Dr. McClellan has explained Mr. Charles Bell's new doctrine of the nerves, to me, the cause is obvious. The total paralysis of the muscles on that side of the face and head* which was immediately discoverable after the tumor had been thus removed, also astonished me not a little, although I now perfectly understand the rationale of that circumstance.

On examining the cavity of the wound, the cartilaginous tube of the ear was found to be exposed down to the auditory process of the temporal bone, and the ligament which ties it to that process was partially divided, so as to give egress to a quantity of blood through the external ear. The articulation of the jaw was partly exposed, so that the interarticular cartilage could be seen behind and above the condyle, on every motion of the joint. The depth of the wound was very great, full two inches and a half from the angle of the jaw towards the posterior fauces, and top of the pharynx. Not a single granule or particle of the gland was left behind. Of this fact we formed a satisfactory

* The auricular muscles, the right half of the occipito-frontalis, the corugator supercilii, the orbicularis palpebrarum, and all the muscles of the nose, cheeks, and lips, of the right side, were totally paralyzed. The levator palpebræ superioris, the muscles of the eyeball, and the muscles of mastication, remained uninjured.

opinion, in as much as the whole of the exposed surface was left clear and dry, after the cessation of the hemorrhage, and could be thoroughly examined by the eye, as well as by the touch.

After having dissected away several diseased lymphatic glands, in the form of small black tubercles, from behind the *sterno-mastoideus*, we dressed the wound with dry lint and adhesive straps, over which was applied a compress and head bandage. The patient was then carried to his bed, having borne the operation with amazing fortitude, and in a sitting posture.

I remained five days and nights with my brother, after the operation, during which time I found occasion to administer no other medicine than a simple effervescing mixture, and a few doses of antimonial solution. The bowels were kept open by injections, and the febrile heat was reduced when the symptomatic fever came on, by ablutions with cold water. No other unfavorable symptoms occurred than a great difficulty of deglutition, and violent pains in the head, which I succeeded in mitigating by the steady application of ice and ice water. In the course of a few weeks after I had returned to Pottsville, I had the pleasure of hearing that our patient had perfectly recovered from the operation, and was actively engaged about his usual business, in his coach manufactory.

It is now eight months since the operation. He is in the enjoyment of perfect health, experiencing no other inconvenience than such as must necessarily result from a deep sunken scar, behind the jaw, which impedes deglutition to some extent, and a total muscular paralysis of the same side of the countenance, which has been produced by the loss of the *portio dura* nerve. He cannot close the eyelids of that side, nor can he laugh without producing excessive distortion towards the opposite side; but his sensibilities are all perfect, both in the eye and over the countenance.

The entire parotid gland has therefore been successfully extirpated, for the second time, on this side of the Atlantic. This operation affords another instance of the skill and intrepidity of American surgeons, and may be placed on a parallel with any that have been recorded in the annals of surgery.

Should any medical gentleman desire to see the patient, I would most respectfully invite then to call on my brother, Mr. Charles Sorber, opposite the falls of the Schuylkill, on the ridge turnpike, four miles from the city of Philadelphia. The tumor has been deposited in the museum of Jefferson College, where it can be examined at any time by those who may desire to receive further satisfaction than I have been able to afford in this detail.*

Philadelphia, Dec. 22, 1829.

---

* The first successful operation for the removal of the parotid gland, was likewise performed in this country, by Dr. McClellan. Its subject was Dr. Graham, now of this city, who has since visited Europe, and satisfied Abernethy, Astley Cooper, and other distinguished surgeons, of the fact of his parotid gland having been removed. A record of the case will be found in the third volume of the American Medical Review and Journal of Philadelphia. We state the fact in this place, because the very possibility of the operation has been denied by high authority—*Ed. N. York Med. and Phys. Jour.*

On the 21st of November, 1830, I visited Mr. Sorber in company with Dr. Drake, and instituted an inquiry into the existing condition of the patient, the results of which may be stated in the following terms:

Mr. Sorber's health has been greatly reduced by gastralgia and other symptoms of dispepsia consequent upon severe and frequently repeated attacks of ague. Still he is able to walk about the fields in the neighborhood of his dwelling, and indulges hopes of ultimate recovery. For the six months immediately succeeding the operation, he enjoyed perfect health; and he attributes his present illness altogether to an obstinate and recurring intermittent, with which he has long been afflicted.

The eyelids contract simultaneously;* and the right eyelid can be depressed considerably—indeed almost sufficient to cover the ball. But the right brow is perfectly destitute of motion, and cannot be depressed, nor can the *corrugator* of that side be moved.

The *buccinator* appears to have acquired some degree of contractile power, and the *levator labii superioris*, has also a slight power of motion. All the other muscles of expression on the injured side, remain totally paralyzed. The masticatory muscles are perfectly natural, and it is impossible to move the *buccinator* and *levator labii superioris*, without, at the same time, depressing that side of the jaw, and drawing it forcibly to the same side.†

He thinks he can bite harder on the sound side, and is certain that he manages his food there as natural. But on the right side he cannot keep the food between his teeth. The lips and inside of his cheek often get between the teeth on the right side, and are unavoidably bitten, in the act of chewing.

The right side is sometimes drier,—after having slept it is a great deal drier than *natural*, and has no moisture at all. He

* He cannot depress the right palpebra when the left is held up by the pressure of the fingers.

† This indicates that it is from the muscular filaments of the inferior maxillary nerve, that the *buccinator* and *levator labii* have begun to recover their contractility.

cannot always feel any difference in eating, but occasionally the left side pours out more fluid during that process.

Sometimes in drinking, the water comes back, both through the mouth and nose. He swallows solids best. He swallows hot articles as well as cold. Sharp drinks, such as brandy and wine, cannot be swallowed readily, and are very apt to fly back from the throat.

Snuff can hardly be felt in the right side of the nostrils, and, on being inhaled, passes into the throat so as to create sickness in the stomach. The left side of the nostril smells naturally, and draws the snuff higher up into its cavity.

There is no difference in the sensibility under the impression of foreign bodies, except that a tender spot exists just over the right zygoma, where the operation gave the most pain. But the right side is a great deal more easily chilled and warmed than the left. In a hot room, the right ear becomes so very hot, that he is often obliged to cool it with a wet rag. In a cold day, the same ear becomes almost frozen.

He does not perspire on the right side either of the face or head, nor has he ever felt any moisture there since the operation.

Before the operation, he states that he had had fever for a long while, and a great difficulty in chewing, and a choking sensation where the lump passed down along the neck. He also vomited every morning, and had very severe pains in the tumor.

During the present winter, I was several times requested to visit Mr. Sorber, whose health was extremely infirm, the symptoms being such as indicated organic abdominal disease; at length he died, and I was requested to make a *post mortem*, the results of which are set forth in the following letter and certificates:

*Philadelphia*, *January* 28, 1831.

To Dr. Drake, M. D., &c.

Sir—Having understood that you were desirous of procuring the detailsof Dr. McClellan's operation on the person of my deceased brother, with a complete history of the case, I transmit to you the accompanying papers. The original account of the operation and its immediate consequences, I published in the last No. of the New-York Med. and Phys. Journal; but as that No. was delayed until the whole concern had expired on the hands of the publishers, I suppose that my paper was not circulated among the profession. You are at liberty, therefore, to publish it in your Western Journal, from my manuscript, with the additional certificates which are now appended to it.

Dr. Runkel, of Germantown, attended my brother during the last two months, and was present with several others at the examination of the body three days after death. I thought, therefore, that a certificate of his opinion of the facts, would render the case more satisfactory to you, especially as he is a physician of great experience and judgment.

Dr. Duncan, of Norristown, was also present, and as he is well known in Philadelphia and in his native state, South Carolina, I send you his certificate, which he annexed to mine directly after the examination was completed.

In conclusion, I will only state, that the patient died of marasmus, from enlarged, or scirrhous pancreas, and obstruction of the *ductus coledochus communis*. The liver did not appear to me to be as light colored, nor as distinctly granulated as natural. The gall bladder was largely distended with thin and watery bile; and two of the mesenteric glands were enlarged to the size of pullets eggs, appeared very dark colored, and adhered to the lower edge of the pancreas. Dr. Samuel McClellan has preserved these parts in the museum of Jefferson College, and states to me, that they partake of the same characters which were manifested in the original tumor of the right parotid gland. The pancreas appear to answer exactly to the description of Mr. Baillie, of a scirrhous enlargement of that gland. The two enlarged mesenteric glands are distinctly melanotic.

It is important that I should state, that there was no glandular enlargement, nor any other morbid appearance in any other part of the body. The right side of the neck and throat was perfectly sound, presenting no trace of disease, except the scar resulting from the operation. With great respect, I am your ob't serv't. JOSEPH E. SORBER.

*Falls of Schuylkill, January* 25, 1831.

I have this moment witnessed the examination of the body and the neck of the late Mr. Charles Sorber, by Drs. Samuel and George McClellan; and certify, that not a vestige of the right parotid gland appeared in any of the recesses behind the ramus of the jaw, or among the small muscles around the styloid process, or in any part with which the parotid is naturally connected. I pronounce, therefore, that the parotid gland has, in this case, been perfectly and completely extirpated, and that his death did not result from any return of the disease in that part of the body. There was disease in the pancreas, gall-bladder, and mesenteric glands, which I consider resulted from the long series of intermittent fevers with which he had been for the last year afflicted. WM. RUNKEL, Jr.

---

I have attended very carefully to the examination of the body of my brother, and am perfectly convinced, that not a vestige of the parotid gland was left behind, in the operation performed by Dr. McClellan. The parts were thoroughly and minutely dissected, and not a single particle of glandular structure could be detected there. What rendered the case more evident was, the dissectoin of the opposite side of the throat, made directly afterwards, which allowed us to contrast the appearances, and prevented all misconception.

J. E. SORBER.

*Falls of Schuylkill, January* 25, 1831.

---

I have just examined the neck of the late Mr. Sorber, and, after a minute inspection of the parts as dissected by Dr. McClellan, (every thing being left *in situ* except the carotid artery, which is taken out with its sheath alone, for the purpose of exhibiting the marks of the ligature placed around it during the operation,) I am fully convinced that the parotid gland was thoroughly extirpated from the right side, by Dr. McClellan, in his operation. The comparison of the appearances on both sides, removes all the doubts from my mind, which I must confess I did entertain before this examination.

ALEX. J. H. DUNCAN, M. D.

*Falls of Schuylkill, January 25th*, 1831.

---

I shall not conceal that I have heard that doubts have been expressed, whether, in this case, the parotid gland was, in reality, extirpated. I trust they will be dissipated from the mind of every candid surgeon, who may read the foregoing certificates.

Respectfully, your ob't serv't,

GEO. McCLELLAN.

